# Depression in Teenager Pregnant Women in a Public Hospital in a Northern Mexican City: Prevalence and Correlates

**DOI:** 10.14740/jocmr2156w

**Published:** 2015-05-08

**Authors:** Cosme Alvarado-Esquivel, Antonio Sifuentes-Alvarez, Carlos Salas-Martinez

**Affiliations:** a Biomedical Research Laboratory, Faculty of Medicine and Nutrition, Juarez University of Durango State, Durango, Mexico; b Mothers and Children’s Hospital, Secretary of Health, Durango, Mexico

**Keywords:** Depression, Pregnancy, Teenagers, Cross-sectional, Epidemiology, Mexico

## Abstract

**Background:**

Very little is known about prenatal depression in teenagers in Mexico. We determined the prevalence and correlates of prenatal depression in teenager women attending a public hospital in Durango City, Mexico.

**Methods:**

We performed a cross-sectional study to assess depression in 181 teenager pregnant women who attended a public hospital for prenatal care. We used a validated Mexican version of the Edinburg postnatal depression scale (EPDS) to screen depression. Women with EPDS scores suggestive of depression were further examined to confirm depression by a psychiatric evaluation using the DSM-IV criteria. Bivariate and multivariate analyses were used to evaluate the prevalence association with socio-demographic, clinical and psychosocial characteristics of the pregnant women.

**Results:**

Of the 181 teenager pregnant women studied, 61 (33.7%) had EPDS equal to or higher than 8 (range 8 - 23), and 37 of them were confirmed to have prenatal depression by the psychiatric evaluation. The general prevalence of prenatal depression in the teenager pregnant women studied was 20.4%. Of the 37 women with depression, 34 suffered from minor depression and three suffered from major depression. Thus, the prevalence of minor and major depression in the women studied was 18.8% and 1.7%, respectively. Multivariate analysis of the socio-demographic, clinical and psychosocial characteristics of the teenager pregnant women showed that prenatal depression was associated with a previous episode of depression during pregnancy (odds ratio (OR) = 6.12; 95% confidence interval (CI): 1.68 - 22.30; P = 0.006), and borderline associations with big fetal size (OR = 9.9; 95% CI: 0.94 - 104.24; P = 0.05) and family problems (OR = 3.83; 95% CI: 0.99 - 14.84; P = 0.05).

**Conclusions:**

Results demonstrate that prenatal depression is common in pregnant teenagers in Durango City, Mexico. The history of an episode of depression during pregnancy should alert physicians for further depression episodes during pregnancy in teenagers. Further research to elucidate the association of prenatal depression with size of the fetus and family problems in pregnant teenagers is needed.

## Introduction

Prenatal depression is a common medical condition reported in many countries [1-3]. Although this condition is highly prevalent, it is still understudied [4], and only few women with major depression seek treatment during pregnancy [5]. Prenatal depression is associated with morbidity in both mothers and offspring [4, 6]. Prenatal depression has been linked to obstetric complications [7], decreased breastfeeding initiation [8], and postpartum psychosis [9]. A meta-analysis of 36 studies showed that depression during pregnancy may be an important risk factor for preterm birth and small-for-gestational-age [10]. Prenatal depression may also lead to postnatal depression [11, 12]. The frequency of depression during pregnancy is also higher than that in the postpartum period [12]. Prevalence of prenatal depression varies substantially among countries [1-3, 13], and this might be due to differences in risk factors in the studied populations. Several risk factors for prenatal depression have been reported including unplanned pregnancy, poverty and marital difficulties [13], domestic violence [13-15], life stress [15], and smoking and alcohol consumption [16]. An important tool to screen depression during pregnancy is the Edinburgh postpartum depression scale (EPDS) [5]. This tool was recently validated for screening depression in teenager pregnant women in a Mexican population [17].

The epidemiology of prenatal depression in teenagers in Mexico has been poorly studied. In a survey of 59 pregnant adolescents in Mexico City, a 39% prevalence of depression was found [18]. Other study of Mexican pregnant women showed that 40% of 45 pregnant adolescents had depressive symptoms [19]. The aims of the present study were to determine the prevalence of depression in teenager pregnant women attended for prenatal care in a public hospital in Durango City, Mexico, and to determine the socio-demographic, clinical and psychosocial characteristics of the teenager pregnant women associated with prenatal depression.

## Materials and Methods

### Study design and women studied

We performed a cross-sectional survey of teenager pregnant women attending their routine prenatal consultations in a public hospital for women and children (Mothers and Children’s Hospital of the Secretary of Health) in Durango City, Mexico. Sampling of women was performed at random from March 2013 to November 2014. Inclusion criteria for enrollment in the study were pregnant women (2 - 9 months of pregnancy) attending their prenatal care in the Mothers and Children’s Hospital, aged 17 years old and younger, and who accepted to participate in the study. Socioeconomic status, residence and occupation of the pregnant women were not restrictive criteria for enrollment. In total, 181 pregnant women were included in the study. They were 13 - 17 years old (mean age: 15.90 ± 0.98 years) and belong to a low socioeconomic status. Pregnant women were evaluated within their 3 - 9 months (median: 7 months) of pregnancy. Of the 181 participants, 154 were in their first pregnancy and 27 were in their 2 - 3 pregnancy.

### Diagnosis of prenatal depression in teenager pregnant women

A validated Mexican version of the EPDS [17] for screening depression in teenager pregnant women was used. We used an EPDS cut-off of 8/9 to screen depression as recommended in the validation study in teenager pregnant women [17]. Pregnant women who scored 8 or higher in the EPDS were interviewed by a psychiatrist to assess major and minor depression by using the DSM-IV criteria [20]. The psychiatrist who examined depression in the teenager pregnant women was blind to the EPDS scores. Both the EPDS and the psychiatric evaluations were performed to each teenager during the same day.

### Socio-demographic, clinical and psychosocial characteristics of the teenager women

The socio-demographic, clinical and psychosocial characteristics of the teenager pregnant women were obtained by a face-to-face interview and data were recorded in a questionnaire. Socio-demographic data included age, occupation, marital status, educational level, birthplace, residence, religion, having a health insurance, age at marriage, and number of marriages. Clinical items were health status, obstetric history, type of obstetric outcome (delivery or cesarean section) of last pregnancy, presence of complications during their last delivery, history of breastfeeding, health status of their last newborn, number of children, history of depression before pregnancy or trauma in live, history of postnatal depression, stress or anxiety before pregnancy, gestational age, number of fetuses in the current pregnancy, knowledge of the fetal sex, fetal sex, a previous depression episode during pregnancy, anxiety or stress during pregnancy, smoking, consumption of alcohol or drugs, and size and health status of the fetus. Psychosocial data including separation from parents at young age, presence of financial or family problems, bad relation with her mother in law, satisfaction with her educational level, support from her couple, relatives, friends or colleagues, support from the Mexican government, unhappiness for the sex of the fetus, unintended pregnancy, bad relation with her couple, living without her couple, abandoned by her couple, violence from her couple, abroad residence of her couple, and satisfaction with her body image from all participants were obtained.

### Statistical analysis

Statistical analyses were performed with the aid of the SPSS version 15.0 software. We used bivariate and multivariate analyses to assess the association between prenatal depression and the characteristics of the teenager pregnant women. We firstly searched for associations by comparing frequencies among groups with the Pearson’s Chi-square and the Fisher exact test (when values were less than 5). As a strategy to include variables in the multivariate analysis we only included variables with a P value equal to or less than 0.05 obtained in the bivariate analysis. Odd ratios (OR) and 95% confidence intervals (CI) were calculated by logistic regression analysis using the Enter method. We used the Hosmer-Lemeshow goodness of fit test to assess the fitness of our regression model. Statistical significance was set at a P value < 0.05.

### Ethical aspects

This study was approved by the Institutional Ethical Committee of the Mothers and Children’s Hospital of the Secretary of Health in Durango City, Mexico. The purpose and procedures of the study were explained to all teenager pregnant women, and a written informed consent was obtained from all of them and their next of kin.

## Results

Of the 181 teenager pregnant women studied, 61 (33.7%) had EPDS equal to or higher than 8 (range 8 - 23), and 37 of them were confirmed to have prenatal depression by the psychiatric evaluation. Therefore, the general prevalence of prenatal depression in the teenager pregnant women studied was 20.4%. Of the 37 women with depression, 34 suffered from minor depression and three suffered from major depression. Thus, the prevalence of minor and major depression in the women studied was 18.8% and 1.7%, respectively. Teenager pregnant women who suffered from prenatal depression were treated with psychotherapy or sertraline.

With respect to the socio-demographic characteristics of the teenager women, bivariate analysis showed that prenatal depression was associated with age, being the highest frequency (56.3%) of prenatal depression at 14 years old (P = 0.001). Other socio-demographic characteristics of women including occupation, marital status, educational level, birthplace, residence, religion, having a health insurance, age at marriage, and number of marriages showed P values > 0.05 by bivariate analysis. Table 1 shows a correlation of depression with the socio-demographic characteristics of the teenager pregnant women.

**Table 1:** Socio-Demographic Characteristics of the Teenager Pregnant Women and Their Association With Depression

Characteristics	No. of women studied	Depression		P value
		No.	%	
Age (years)				
13	1	0	0	0.001
14	16	9	56.3	
15	42	6	14.3	
16	64	7	10.9	
17	58	15	25.9	
Occupation				
Laborer	3	0	0	1.00
Non-laborer	178	37	20.8	
Marital status				
Married	25	5	20	0.65
Single	43	11	25.6	
Living together	113	21	18.6	
Education				
1 - 6 years	1	0	0	0.19
7 - 12 years	11	0	0	
> 12 years	169	37	21.9	
Birthplace				
Durango State	181	37	20.4	
Other Mexican State	0	0		
Residence place				
Durango State	177	37	20.9	0.30
Other Mexican State	4	0	0	
Residence area				
Urban	152	32	21.1	0.54
Suburban	17	4	23.5	
Rural	12	1	8.3	
Religion				
Yes	174	37	21.3	0.34
No	7	0	0	
Health insurance				
Yes	176	36	20.5	1.00
No	5	1	20	
Age at marriage				
11 - 14	39	10	25.6	0.27
15 - 17	119	21	17.6	
No. of marriages				
None	156	32	20.5	0.46
One	20	5	20	
Two	5	0	0	

Of the clinical characteristics, bivariate analysis showed that depression was associated with depression (P = 0.01) and anxiety (P = 0.01) before pregnancy, trimester of pregnancy (P = 0.02), a previous episode of depression during pregnancy (P < 0.001), anxiety (P < 0.001) and stress (P = 0.004) during pregnancy, and fetal size (P = 0.001). Other clinical characteristics of the teenager women including health status, obstetric history, type of obstetric outcome of last pregnancy, presence of complications during their last delivery, history of breastfeeding, health status of their last newborn, number of children, history of trauma or postnatal depression, stress before pregnancy, number of fetuses in the current pregnancy, knowledge of the fetal sex, fetal sex, smoking, consumption of alcohol or drugs, and health status of the fetus showed P values > 0.05 by bivariate analysis. Table 2 shows a selection of clinical characteristics in the teenager women and their association with prenatal depression.

**Table 2:** Bivariate Analysis of a Selection of Clinical Characteristics of the Teenager Pregnant Women and Their Association With Depression

Characteristics	No. of women studied	Depression		P value
		No.	%	
Health status				
Ill	8	0	0	0.36
Healthy	173	37	21.4	
No. of pregnancies				
One	154	34	22.1	0.41
Two	26	3	11.5	
Three	1	0	0	
No. of deliveries				
None	174	35	20.1	0.63
One	7	2	28.6	
Cesarean sections				
Yes	6	0	0	0.34
No	175	37	21.1	
Miscarriages				
Yes	13	1	7.7	0.47
No	168	36	21.4	
Outcome of last pregnancy				
Delivery	7	2	28.6	0.46
Cesarean section	6	0	0	
Complication in last pregnancy				
Yes	2	1	50	0.42
No	11	2	18.2	
Depression before pregnancy				
Yes	40	14	35	0.01
No	140	23	16.4	
Trauma in life				
Yes	18	6	33.3	0.21
No	162	31	19.1	
History of postpartum depression				
Yes	15	6	40	0.09
No	126	25	19.8	
Stress before pregnancy				
Yes	47	13	27.7	0.18
No	130	24	18.5	
Anxiety before pregnancy				
Yes	41	14	34.1	0.01
No	136	23	16.9	
Trimester of pregnancy				
First	8	4	50	0.02
Second	61	16	26.2	
Third	112	17	15.2	
Know the fetal sex				
Yes	103	19	18.4	0.36
No	75	18	24	
Fetal sex				
Male	59	10	16.9	0.68
Female	45	9	20	
Previous depression during pregnancy				
Yes	48	24	50	< 0.001
No	133	13	9.8	
Anxiety during pregnancy				
Yes	48	19	39.6	< 0.001
No	133	18	13.5	
Stress during pregnancy				
Yes	74	23	31.1	0.004
No	106	14	13.2	
Smoking				
Yes	10	3	30	0.43
No	170	34	20	
Alcohol consumption				
Yes	11	3	27.3	0.69
No	169	34	20.1	
Drug abuse				
Yes	5	1	20	1.00
No	175	35	20	
Fetal size				
Small	6	1	16.7	0.001
Normal	140	29	20.7	
Big	4	4	100	

With respect to the psychosocial characteristics of the teenager pregnant women, bivariate analysis showed that depression was associated with financial (P = 0.01) and family (P = 0.01) problems, bad relation with her couple (P = 0.003), living without her couple (P = 0.01), ever abandoned by her couple (P = 0.008), and violence from her couple (P = 0.02). Other psychosocial characteristics of the women including separation from parents at young age, bad relation with her mother in law, satisfaction with her educational level, support from her couple, relatives, friends or colleagues, support from the Mexican government, unhappiness for the sex of the fetus, unintended pregnancy, abroad residence of her couple, and satisfaction with her body image showed P values > 0.05 by bivariate analysis. Table 3 shows a correlation of prenatal depression with the psychosocial characteristics of the teenager women. Regression analysis (Table 4) of the socio-demographic, clinical and psychosocial characteristics of the teenager pregnant women with P values < 0.05 by bivariate analysis showed that prenatal depression was associated with a previous episode of depression during pregnancy (OR = 6.12; 95% CI: 1.68 - 22.30; P = 0.006), and borderline associations with big fetal size (OR = 9.9; 95% CI: 0.94 - 104.24; P = 0.05) and family problems (OR = 3.83; 95% CI: 0.99 - 14.84; P = 0.05). The result of the Hosmer-Lemeshow test (P = 0.47) indicated an acceptable fit of our regression model.

**Table 3:** Bivariate Analysis of Psychosocial Characteristics of the Teenager Pregnant Women and Their Association With Depression

Characteristics	No. of women studied	Depression		P value
		No.	%	
Separated from parents at young age				
Yes	55	14	25.5	0.28
No	125	23	18.4	
Financial problems				
Yes	38	13	34.2	0.01
No	143	24	16.8	
Family problems				
Yes	21	9	42.9	0.01
No	160	28	17.5	
Bad relation with mother in law				
Yes	32	6	18.8	1.00
No	142	29	20.4	
Satisfaction with educational level				
Yes	103	21	20.4	1.00
No	77	16	20.8	
Support from her couple				
Yes	152	28	18.4	0.09
No	28	9	32.1	
Support from relatives, friends, colleagues				
Yes	155	31	20	0.71
No	26	6	23.1	
Support from the government				
Yes	53	11	20.8	0.94
No	128	26	20.3	
Happy with the fetal sex				
Yes	129	30	23.3	0.53
No	28	5	17.9	
Desired pregnancy				
Yes	117	20	17.1	0.13
No	64	17	26.6	
Relation with her couple				
Good	143	23	16.1	0.003
Bad	30	12	40	
Live with her couple				
Yes	126	20	15.9	0.01
No	45	15	33.3	
Ever abandoned by her couple				
Yes	47	16	34	0.008
No	127	20	15.7	
Violence from her couple				
Yes	22	9	40.9	0.02
No	151	27	17.9	
Couple living abroad				
Yes	5	0	0	0.58
No	167	35	21	
Satisfied with her body image				
Yes	167	31	18.6	0.06
No	12	5	41.7	

**Table 4:** Multivariate Analysis of Selected Characteristics of the Teenager Pregnant Women and Their Association With Depression

Characteristic	Odds ratio	95% confidence interval	P value
Age	1.07	0.66 - 1.74	0.75
Depression before pregnancy	1.18	0.33 - 4.10	0.79
Anxiety before pregnancy	0.91	0.24 - 3.44	0.89
Trimester of pregnancy	0.58	0.25 - 1.35	0.21
Previous depression during pregnancy	6.12	1.68 - 22.30	0.006
Anxiety during pregnancy	1.04	0.28 - 3.82	0.94
Stress during pregnancy	0.94	0.25 - 3.46	0.93
Fetal size	9.9	0.94 - 104.24	0.05
Financial problems	1.06	0.31 - 3.65	0.91
Family problems	3.83	0.99 - 14.84	0.05
Bad relation with her couple	1.04	0.22 - 4.81	0.95
Living without her couple	0.62	0.15 - 2.44	0.49
Ever abandoned by her couple	0.99	0.24 - 4.13	0.99
Violence from her couple	1.9	0.53 - 6.74	0.31

## Discussion

The prevalence and correlates of depression in teenager pregnant women in Mexico have been scantily studied. Prenatal depression is a frequent, albeit a hitherto neglected medical condition in Mexico. In the present work, we determined the prevalence and correlates of depression in teenager pregnant women who were attended for routine prenatal care in a Mothers and Children’s Public Hospital in Durango City, Mexico. We found a 20.4% prevalence of prenatal depression in the teenager women studied. This prevalence is lower than the 32.5% frequency of depressive symptoms in adolescent pregnant women during their second trimester of pregnancy found in a national survey in Mexico [21], and the 39% prevalence of depression in pregnant adolescents in Mexico City [18]. However, comparison of these prevalences of prenatal depression in pregnant teenagers should be interpreted with care since difference tools to evaluate depression among the studies were used. We used the EPDS followed by psychiatric confirmation using the DSM-IV criteria for depression. Whereas the Center for Epidemiological Studies Depression Scale was used in the national survey [21], and the Beck Depression Inventory in the study in Mexico City [18]. We are not aware of prevalence studies in teenager pregnant women using the EPDS in Mexico or other country. Therefore, the prevalence of prenatal depression found in the present study cannot be fairly compared with those reported in other studies using a number of several tools for testing depression. In an international context, the prevalence of prenatal depression found in teenagers in Durango is comparable with the 20.8% prevalence of depression found in 120 pregnant adolescents in Brazil by using the hospital anxiety and depression scale [22]. In contrast, the prevalence of prenatal depression in Mexican teenagers found in this study is lower than the 56.6% prevalence of depressive symptoms found in pregnant adolescents in Guayaquil, Ecuador by using the 10-item Center for Epidemiological Studies Short Depression Scale [23]. Concerning major depression, the prevalence of major depression found in our study (1.7%) is lower than the 17.8% prevalence of major depression disorders found in pregnant teenagers in Brazil [24].

We sought correlates of prenatal depression in pregnant teenagers. Logistic regression of socio-demographic, clinical and psychosocial characteristics of the pregnant teenagers showed that depression was associated with a previous episode of depression during pregnancy. This result suggests that episodes of depression in pregnant teenagers may be repetitive. There is poor knowledge about the frequency of repetitive episodes of depression during pregnancy, and further research to quantify the number and duration of depressive episodes during pregnancy is needed. Concerning the borderline association of prenatal depression with big fetal size, we are not aware of a previous report about an association of fetal size with depression in pregnant women. It is unclear why pregnant teenagers with big fetal size had a higher prevalence of depression than those with normal or small fetal size. It is possible that pregnant women with a big fetus are worried for the health of their fetus and the likelihood of having any obstetric complication. Women giving birth to a child with very large head circumference are more likely to have prolonged labors, signs of fetal distress, and maternal distress than are women giving birth to a child with average head size [25]. In addition, these big fetuses may cause assisted vaginal births and emergency cesarean sections [25]. With respect to the borderline association of prenatal depression with family problems in pregnant teenagers found in the present study, our result conflicts with that found in a study of 222 pregnant women in Lima, Peru, where researchers found that family problems did not influence the prevalence of depression [26]. However, the Peruvian study was performed in women aged 16 - 42 years old whereas we studied only teenager women. There is a poor knowledge about the association of family problems with prenatal depression in teenagers. This factor has rather been studied in adult women in the postpartum period. In a previous study of women in Durango, we found that postnatal depression was associated with family problems [11]. The identification of family problems as an important factor for both prenatal and postpartum depression in the region warrants for further multidisciplinary research searching for optimal preventive measures against this factor to reduce the rate of depression in women.

### Conclusions

Results demonstrate that prenatal depression is common in pregnant teenagers in Durango City, Mexico. The history of an episode of depression during pregnancy should alert physicians for further depression episodes during pregnancy in teenagers. Further research to elucidate the association of prenatal depression with size of the fetus and family problems in pregnant teenagers is needed.
